# Ocular pulse amplitude and retina nerve fiber layer thickness in migraine patients without aura

**DOI:** 10.1186/s12886-015-0180-2

**Published:** 2016-01-04

**Authors:** Semra Acer, Attila Oğuzhanoğlu, Ebru Nevin Çetin, Nedim Ongun, Gökhan Pekel, Alper Kaşıkçı, Ramazan Yağcı

**Affiliations:** Department of Ophthalmology, Kinikli Kampusu, Pamukkale University, Denizli, TR 20100 Turkey; Department of Neurology, Kinikli Kampusu, Pamukkale University, Denizli, Turkey

**Keywords:** Cerebrovascular disease, Ocular pulse amplitude, Choroidal perfusion, Nerve fiber, Retinal imaging

## Abstract

**Background:**

To evaluate the ocular pulse amplitude (OPA), the posterior pole asymmetry analysis (PPAA), the peripapillary retinal nerve fiber layer (RNFL) thickness, the ganglion cell layer (GCL) thickness, macular thickness and visual field testing in migraine patients without aura.

**Methods:**

In this prospective, cross-sectional and comparative study 38 migraine patients and 44 age and sex matched controls were included. OPA was measured by dynamic contour tonometry (DCT), PPAA, RNFL, GCL and macular thickness were measured by Heidelberg Spectral Domain Optical Coherence Tomography (SD-OCT) and standard perimetry was performed using the Humphrey automated field analyzer.

**Results:**

The difference in OPA was not statistically significant between the two groups (p ≥ 0.05). In the PPAA there was no significant difference between two hemispheres in each eye (p ≥ 0.05). The RNFL thickness was significantly reduced in the temporal and nasal superior sectors in the migraine group (p ≤ 0.05). The GCL and macular thickness measurements were thinner in migraine patients but the difference between groups was not statistically significant (p ≥ 0.05). There was no correlation between RNFL, GCL, macular thickness measurements and OPA values. There was no significant difference in the mean deviation (MD) and pattern standard deviation (PSD) between the two groups (p ≥ 0.05).

**Conclusions:**

Migraine patients without aura have normal OPA values, no significant asymmetry of the posterior pole and decreased peripapillary RNFL thickness in the temporal and nasal superior sectors compared with controls. These findings suggest that there is sectorial RNFL thinning in migraine patients without aura and pulsative choroidal blood flow may not be affected during the chronic course of disease.

## Background

Migraine is a form of headache that has been regarded as a *neurovascular* disorder. The theories about its mechanism do not provide satisfactory clarification and the underlying pathophysiology still remains unclear. Recent studies declared that neural events trigger vascular dilatation, which aggravates the pain and results in further nerve activation [1]. Migraine has several ocular manifestations including visual disturbances during attacks [2], visual field defects [3], reduction in retinal nerve fiber layer (RNFL) thickness [4, 5], and normal tension glaucoma [6]. Central retinal artery occlusion and ischemic optic neuropathy were also reported previously, which may indicate possible ocular vascular involvement as well as central vascular system in migraine [7–9]. Vasospastic events and ocular involvement were reported mostly in migraine patients with aura [1].

Ocular pulse amplitude (OPA), which is described as the difference between systolic and diastolic intraocular pressure (IOP), is accepted as an indirect indicator of choroidal perfusion [10]. It is measured by dynamic contour tonometry, which is a contact tonometry providing intraocular pressure and OPA simultaneously and was suggested as a diagnostic tool for diseases in which choroidal perfusion changes might occur [10, 11].

The posterior pole asymmetry analysis (PPAA) is a new method determining the hemisphere asymmetry of the macula by comparing the thickness of the two hemispheres on a map, which are divided into equal cells. It is reported that PPAA could show early glaucomatous damage before it could be documented by the optic disc analysis [12]. Therefore, there is a current trend towards investigating the change in PPAA in various ocular disorders in which retinal ganglion cell or axonal damage may occur.

The majority of migraine patients (85 %) have migraine without aura [1]. Recently intracerebral vascular alterations were reported in patients without aura which may support that vascular dysregulation is an integral mechanism of the migraine pathophysiology [13]. Therefore we conducted this study to evaluate the ocular involvement in migraine patients without aura, using OPA, PPAA, peripapillary RNFL thickness, GCL thickness, macular thickness and visual field test.

## Methods

This prospective observational cross sectional study was approved by the Ethical Committee of Pamukkale University and adhered to the tenets of the Declaration of Helsinki. The study was conducted in the Department of Ophthalmology and the Department of Neurology in our university. All participants provided informed written consent to participate in the study. The diagnostic criteria for migraine without aura was based on the international Classification of Headache Disorder (ICHD-II) [14];

*At least five of the following criteria need to be met:*

- Treated or untreated headache attacks lasting 4 to 72 h.

*Headache has at least two of the following characteristics:*

- Moderate or severe pain intensity.
- Pulsating quality.
- Unilateral location.
- Aggravation by physical activity or causing avoidance of routine physical activity (eg, walking).

*During a headache at least one of the following:*

- Photophobia and phonophobia.
- Nausea and/or vomiting.
- Symptoms not associated with another disorder.

### Study population

The participants were 38 migraine patients without aura and 44 age and sex matched controls.

The exclusion criteria for both groups were;

- Age ≥ 45 (to exclude age related changing)
- Best corrected visual acuity (BCVA) with Snellen chart < 1.0 (in decimals, any level of visual impairment was excluded)
- Having spherical or cylindrical refractive error ≥ 2D
- Having any ocular disease that could affect the RNFL thickness (glaucoma etc.)
- Previous ocular inflammation or surgery
- Any systemic or neurologic disorder
- Chronic drug use including migraine prophylactic agents which could influence ocular blood flow and RNFL thickness.

### Examination techniques

All patients underwent detailed ophthalmic examination including visual acuity, intraocular pressure with non contact tonometry, ocular motility, anterior and posterior segment evaluations. OCT measurements were performed first, as DCT may cause transient corneal opacifications. Visual field testing was performed on a separate day.

OPA was measured by dynamic contour tonometry (DCT, Ziemer Ophthalmic Systems, Port, Switzerland) after applying a topical anaesthetic eye drop. The DCT is attached to a slit lamp, like classic applanation tonometers, giving a medium value of 100 IOP measurements per second and determines OPA, by providing dynamic IOP measurements. The mean value of two reliable measurements (Q1, Q2, Q3, as recommended by the manufacturer) was used.

**Fig. 1:**
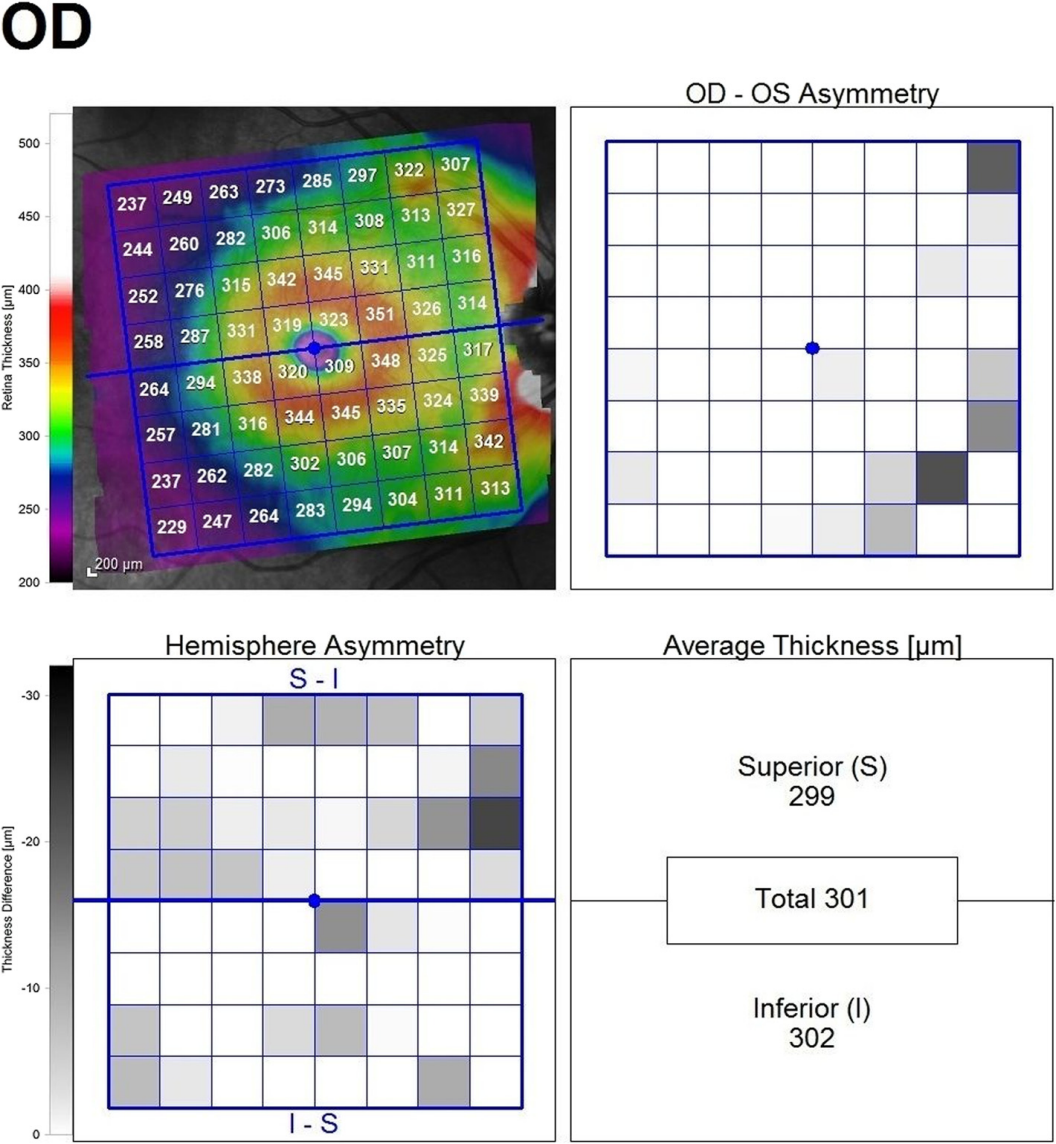
Topographic measurement of PPAA. This migraine patient has a few gray cells, but no black cells and no significant hemisphere asymmetry

**Fig. 2:**
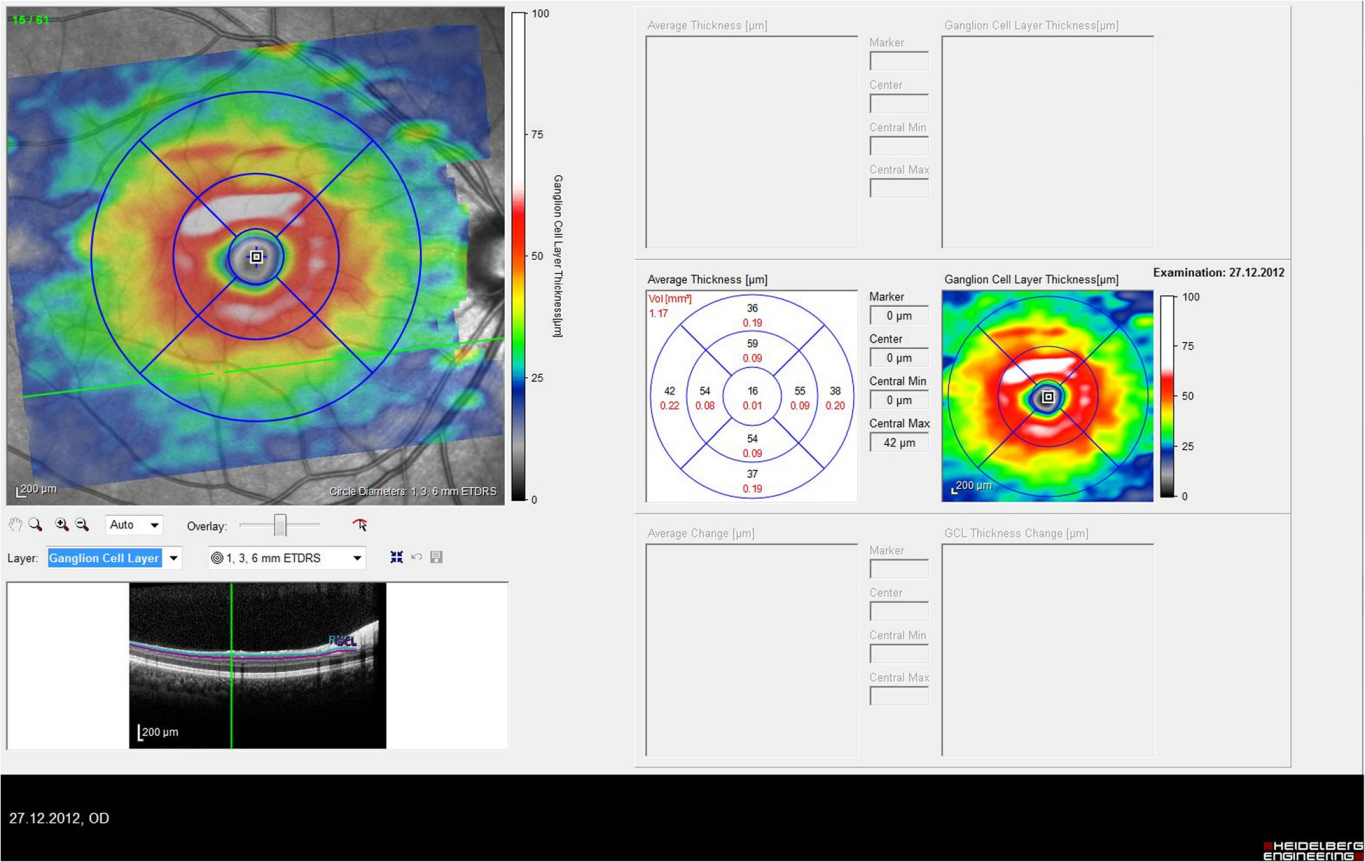
Topographic measurement of GCL thickness of a patient in the study

Without pupillary dilatation, the PPAA, peripapillary RNFL thickness, GCL thickness and macular thickness were analysed with the Heidelberg Spectral Domain Optical Coherence Tomography (SD-OCT, Heidelberg Engineering, Dossenheim, Germany). The images that individual retinal layers could be identified were used. Asymmetry analysis of the posterior pole was evaluated with a map which compares the superior to inferior hemispheres (hemisphere asymmetry) for each eye. See Fig. 1 for asymmetry analysis of the posterior pole. One hemisphere includes 32 cells and each cell has an equivalent in the opposite hemisphere. The difference in retinal thickness between the two equivalent cells are indicated with colors changing from white to black. A black cell means that the difference in retinal thickness is ≥30 μm. For cell to cell comparison between two hemispheres, two or more consecutive black cells were taken into consideration and recorded as asymmetry positivity. The peripapillary RNFL thickness was evaluated in six sectors including temporal inferior, temporal, temporal superior, nasal superior, nasal, nasal inferior. GCL thickness was measured on a central macular map which was automatically divided into ETDRS macular fields with a diameter of 1 mm (central sector),3 mm (internal sectors) and 6 mm (external sectors) in horizontal and vertical planes. The mean GCL thickness of central macular field with a diameter of 3 mm was estimated as the mean GCL thickness in each patient. See Fig. 2 for topographic measurement of GCL thickness.

Standard perimetry was performed using the central 30-2 threshold algorithm program on a Humphrey automated field analyser (HFA, CarlZeiss Inc, Dublin, California). The criteria for visual field reliability included <20 % fixation loss and < 20 % false positive and negative ratios. The test was repeated in patients with low reliability. The mean deviation (MD) and pattern standard deviation (PSD) values were recorded.

Randomly one eye of each participant was selected for the comparison of the parameters between the migraine and the control group.

### Statistical analysis

Statistical analysis was performed by SPSS statistical software 17.0 (SPSS Inc., Chicago, IL). Independent samples t test, Pearson’s correlation, and chi-square test were used for statistical analysis. Descriptive statistics were stated as means ± SD. A value of p ≤ 0.05 has been accepted as significant.

## Results

The mean age was 30.17 ± 11.15 years in the migraine group, and 28.75 ± 7.06 years in the control group (*p* = 0.42). There were 35 females and 3 males in the migraine group and 40 females and 4 males in the control group (*p* = 0.84). The two groups were equivalent in terms of ethnicity. There was no difference in the refractive status between groups (*p* = 0.77). The mean number of migraine attacks per month was 4.30 ± 2.50. The mean length of migraine history was 3.72 ± 3.16.months.

**Fig. 3:**
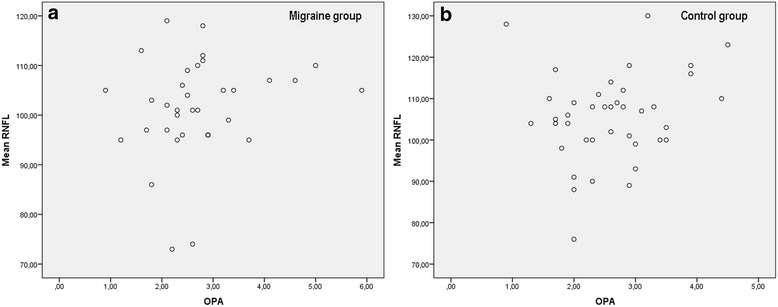
Correlation between OPA and mean RNFL thickness in the (**a**) migraine and (**b**) control groups

The OPA value was 2.78 ± 1.02 mmHg and 2.61 ± 0.81 mmHg in the migraine and the control group respectively (*p* = 0.48). The correlation between OPA and the mean RNFL thickness in the migraine and the control group is shown in Fig. 3. There was no correlation between OPA and mean RNFL thickness (*r* = 0.19, *p* = 0.26), mean GCL thickness (*r* = 0.06, *p* = 0.56), macular thickness (*r* = 0.23, *p* = 0.16), MD (*r* = 0.01, *p* = 0.95) and PSD (*r* = -0.37, *p* = 0.12) in the migraine group.

The difference in PPAA between the two groups was not statistically significant (*p* = 0.83). The PPAA of a migraine patient is shown in Fig. 2. Table 1 shows the macular thickness measurements and Table 2 shows the GCL thickness measurements in both groups. RNFL thickness was significantly lower in the temporal and the nasal superior sectors in migraine group as shown in Table 3. Illness duration or frequency did not show a correlation with OPA, RNFL thickness, macular thickness, GCL thickness, MD or PSD.

**Table 1:** Macular thickness measurements in migraine and control group

Macular thickness (μm)	Migraine (*n* = 38)	Control (*n* = 44)	*p* value
Macular thickness	296.80 ± 12.47	299.02 ± 12.86	0.46
Superior hemifield thickness	296.83 ± 12.92	297.73 ± 13.63	0.77
Inferior hemifield thickness	297.58 ± 12.55	299.78 ± 12.90	0.47

**Table 2:** GCL thickness measurements in the migraine and control groups

GCL thickness (μm)	Migraine (*n* = 38)	Control (*n* = 44)	*p* value
Mean GCL thickness	42.84 ± 4.14	44.53 ± 3.81	0.07
Central sector GCL thickness	12.79 ± 3.42	13.00 ± 3.04	0.78

*GCL* Ganglion cell layer

**Table 3:** RNFL thickness measurements in the migraine and control groups

RNFL Thickness (μm)	Migraine (*n* = 38)	Control (*n* = 44)	*p* value
Mean RNFL	102.39 ± 8.91	105.63 ± 10.64	0.15
Temporal inferior	151.64 ± 17.95	157.89 ± 20.88	0.16
Temporal	71.47 ± 10.29	77.93 ± 11.34	**0.01**
Temporal superior	142.38 ± 13.50	143.27 ± 22.10	0.83
Nasal superior	103.94 ± 13.67	112.25 ± 22.02	**0.04**
Nasal	80.97 ± 13.73	76.93 ± 12.16	0.17
Nasal inferior	119.08 ± 19.68	119.48 ± 22.79	0.93

*RNFL* retinal nerve fiber layer

The bold p values were statically significant

**Fig. 4:**
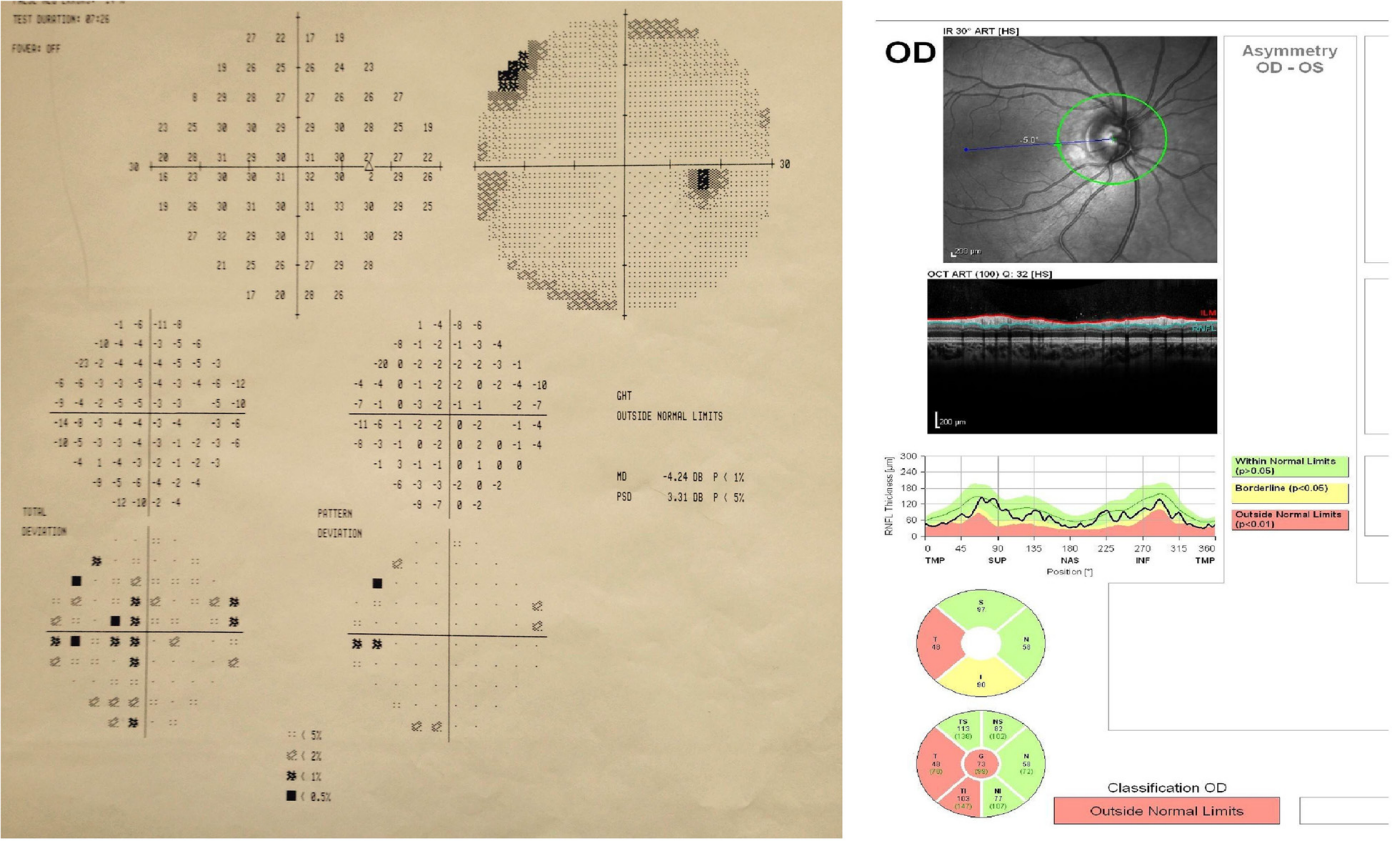
Visual field defects and sectorial RNFL thinning of a migraine patient in the study

In the visual field testing; the MD was -2.90 ± 1.81 dB and -2.32 ± 1.86 dB, the PSD was 2.01 ± 0.55 dB and 1.90 ± 0.73 dB in the migraine and the control groups, respectively. The difference between the two groups was not statistically significant (*p* = 0.24 for MD and *p* = 0.51 for PSD). In the migraine group there was no correlation between MD and RNFL in any sector but there was a negative correlation between PSD and mean RNFL (*p* = 0.01, *r* = -0.54) and in some sectors including temporal superior (*p* = 0.03, *r* = -0.48) nasal superior (*p* = 0.04, *r* = -0.45), nasal inferior (*p* = 0.02, *r* = -0.52). Fig. 4 shows visual field defects and sectorial RNFL thinning in a migraine patient.

## Discussion

Our findings suggest that the choroidal perfusion assessed by OPA, is not significantly different between the migraine patients and the controls. Similarly, PPAA is not significantly different between the groups. However, there is significant sectoral RNFL thinning which correlates with PSD –an indicator of localized visual field defects- but not with OPA in migraine patients without aura.

Recent studies have reported choroidal and retinal involvement in migraine patients [4, 5, 15]. Using colored doppler ultrasonography, reduction of blood flow has been shown in both the central retinal artery and posterior ciliary artery in migraine patients without aura compared to healty subjects [16]. Decrease in choroidal blood flow by stimulation of choroidal sympathetic supply [17, 18], amaurosis fugax in young migraneurs [19] and pigmentary changes on fundus examination following attacks [20] were suggested as signs of affected choroidal circulation in migraine. OPA reflects pulsatile variations in IOP which corresponds to the blood volume (mostly from the choroidal bed) pumped into the eye during each cardiac cycle [10, 11]. Therefore, OPA was suggested as an indirect indicator of choroidal blood flow and has been investigated in several disorders with vascular origin [10, 21, 22]. Decreased OPA values were reported in patients with carotid artery stenosis, uveitis, normal tension glaucoma and primary open angle glaucoma [10, 23]. We assessed the choroidal blood flow by measuring OPA and could not find a significant difference in OPA between the migraine group and controls. The result of this study shows that there is a possibility that the pulsative choroidal blood flow is not affected in patients without aura. This outcome does not rule out the involvement of choroidal perfusion but rather shows that the pulsative choroidal blood flow is not permanently compromised in this subgroup.

If migraine might affect the peripapillary RNFL, it might also affect the GCL. The GCL is thickest at macula and the RNFL is thickest at peripapillary region [24]. Although glaucoma damages both GCL and RNFL, the peripapillary RNFL thickness has been shown to be more sensitive in the detection of glaucoma [25]. In recent years measurements of inner macular layers consisting of GCL, RNFL and the inner plexifom layer and macular asymmetry has been reported as an early predicting sign of glaucoma damage [12, 24]. Heidelberg SD-OCT has a new macular map consisting of 64 cells which may give a chance of a particular macular hemisphere analysis [12, 26]. PPAA may show the early damage of the nerve fiber layer, which usually starts in one hemisphere, by comparing the cells of two hemispheres. But in the progressive retinal fiber layer loss, an asymmetry is not usually present as advanced fiber loss includes both hemispheres [12]. Performing this test in migraneurs may not only provide data about macular nerve damage, but also may help detect an early onset of normal tension glaucoma in these patients. There was no significant difference between the two groups with cell to cell comparison. We also compared GCL and macular thickness in the two groups. Both GCL and macular thickness measurements were thinner in migraine patients but the difference was not statistically significant. Reduction in macular thickness with accompanying peripapillary RNFL thinning was documented in a migraine patient [27]. Ekinci et all reported decreased GCL thickness measurements in migraine patients and thinning in GCL was significant in migraine patients with aura [15]. Our results may reflect that nerve damage is not significant in the macular area in migraine patients without aura.

RNFL thickness was significantly lower in the temporal and nasal superior sectors. Inner retinal layers including RNFL and GCL are supplied by retinal vessels and outer retinal layers are supplied by choroidal vessels [28]. In contrast to choroidal vessels, retinal vessels have no autonomic innervations [29]. During an acute attack, the neural events result in dilation of blood vessels that cause nerve activation and vasoactive peptides being released into the extracerebral circulation. The vasoactive substances initiate a neurogenic inflammation and these neurovascular events are assumed to be the cause of pain [1]. These vasoactive substances may also reduce the ocular blood flow and adequate oxygen may not be provided to the retinal neural cells, which may result in cell deterioration. Ekinci et al showed that RNFL thickness was thinner in migraine patients with aura, compared with patients without aura and healthy controls [15]. Gipponi and Martinez reported a reduction in RNFL thickness in migraine patients with and without aura [4, 5]. In this study we reported decreased RNFL thickness in patients without aura which may suggest that the neurovascular pathway underlying the migraine may play a role in reducing RNFL thickness independent of aura. Previous studies have reported segmental thinning of the temporal and superior RNFL which is consistent with our results [4, 5]. Finding RNFL thinning in similar sectors may be accidental or may be specific for migraine disease. There was no correlation between RNFL thinning and illness duration or frequency, however to exclude patients with anti-migraine drug use, we had to include patients who had newly diagnosed migraine or had short illness duration. Therefore, a long term follow-up is needed to observe the course of thinning.

In the visual field testing, both MD and PSD were worse in the migraine group, but the difference was not statistically significant and no correlation was found between MD, PSD and illness duration or frequency. Unexpectedly, MD was -2.32 ± 1.86 dB in controls, which might be a result of the practice and learning effect. Migraine patients had localized superior and inferior hemifield visual field losses (25 %), most of which correlated with the thinning of the corresponding RNFL quadrants. There was no correlation between MD and RNFL in any sector, but there was a negative correlation between PSD and the mean RNFL, temporal superior, nasal superior and nasal inferior sectors. General reduction of sensitivity was present in 10 % of migraine patients. Different forms of visual field defects, most of which were present in the midperipheral field have been reported in migraneurs, reflecting a precortical region [30, 31]. Cortical visual field defects are not common in migraine patients [32]. Because of the relationship between normal tension glaucoma and migraine, migraine patients who have RNFL thinning and visual field defects, even though they have no glaucomatous cupping, should be carefully monitored, in terms of glaucoma.

Our study has several limitations. Firstly; OPA measurements could not be obtained during migraine attacks because most of the patients were not able to show up during their attacks. Secondly; choroidal thickness with enhanced deep imaging OCT should have been measured and the results should have been compared between groups. Lastly; It would be better to evaluate the choroidal doppler ultrasound and correlate it with OPA findings.

## Conclusions

In migraine patients without aura, the pulsative choroidal blood flow is not affected, at least between attacks. There is significant sectorial RNFL thinning which correlates with localized visual field defects. This finding may indicate an axonal insult and the necessity of monitoring RNFL and visual field testing in migraine patients without aura.

## Abbreviations

BCVA: best corrected visual acuity
DCT: dynamic contour tonometry
GCL: Ganglion cell layer
ICHD: international classification of headache disorder
MD: mean deviation
OCT: optical coherence tomography
OPA: ocular pulse amplitude
PPAA: posterior pole asymmetry analysis
PSD: pattern standard deviation
RNFL: retinal nerve fiber layer
